# Glycogen Granules Are Degraded by Non-Selective Autophagy in Nitrogen-Starved *Komagataella phaffii*

**DOI:** 10.3390/cells13060467

**Published:** 2024-03-07

**Authors:** Nimna V. Wijewantha, Ravinder Kumar, Taras Y. Nazarko

**Affiliations:** 1 Department of Biology, Georgia State University, Atlanta, GA 30303, USA; nwellalawijewantha1@gsu.edu; 2 Section of Molecular Biology, Division of Biological Sciences, University of California, San Diego, La Jolla, CA 92093, USA

**Keywords:** autophagy, Glg1, glycogen, glycogen granules, glycophagy, *Komagataella phaffii*, non-selective autophagy, *Pichia pastoris*, selective autophagy, yeast

## Abstract

Autophagy was initially recognized as a bulk degradation process that randomly sequesters and degrades cytoplasmic material in lysosomes (vacuoles in yeast). In recent years, various types of selective autophagy have been discovered. Glycophagy, the selective autophagy of glycogen granules, is one of them. While autophagy of glycogen is an important contributor to Pompe disease, which is characterized by the lysosomal accumulation of glycogen, its selectivity is still a matter of debate. Here, we developed the *Komagataella phaffii* yeast as a simple model of glycogen autophagy under nitrogen starvation conditions to address the question of its selectivity. For this, we turned the self-glucosylating initiator of glycogen synthesis, Glg1, which is covalently bound to glycogen, into the Glg1-GFP autophagic reporter. Our results revealed that vacuolar delivery of Glg1-GFP and its processing to free GFP were strictly dependent on autophagic machinery and vacuolar proteolysis. Notably, this process was independent of Atg11, the scaffold protein common for many selective autophagy pathways. Importantly, the non-mutated Glg1-GFP (which synthesizes and marks glycogen) and mutated Glg1^Y212F^-GFP (which does not synthesize glycogen and is degraded by non-selective autophagy as cytosolic Pgk1-GFP) were equally well delivered to the vacuole and had similar levels of released GFP. Therefore, we concluded that glycogen autophagy is a non-selective process in *K. phaffii* yeast under nitrogen starvation conditions.

## 1. Introduction

Glycogen is a primary storage form of glucose. It is an efficient source of energy that is readily available for decomposition to glucose. Glycogen is associated with various enzymes and regulatory proteins involved in its metabolism, resulting in the glycogen-protein complex called the glycogen granule (GG) or glycosome [1]. The initiation of glycogen synthesis occurs through the action of glycogenin, the central priming protein, which catalyzes the auto-glucosylation of its conserved tyrosine residue. This covalent binding process results in the production of a short-chain oligomer of approximately 12 glucose residues linked by α-1,4-glycosidic bonds. Subsequently, these short-chain oligosaccharides covalently bound to glycogenin molecules are further extended via α-1,4-glycosidic linkages and spread out via α-1,6-glycosidic bonds at branch points by other enzymes of the glycogen biosynthesis pathway [2,3].

In *Saccharomyces cerevisiae*, there are two glycogenins, Glg1 and Glg2, which emerged after the whole genome duplication [4,5]. In contrast, another popular yeast model, *Komagataella phaffii* (formerly *Pichia pastoris*), has only one glycogenin (called Glg1 here), which exhibits sequence similarity with *S*. *cerevisiae* proteins (Figure S1). Notably, *S*. *cerevisiae* glycogenins contain a conserved tyrosine residue, Tyr^230^ in Glg1 and Tyr^232^ in Glg2, which forms the glucose-1-*O*-tyrosyl linkage when subjected to incubation with UDP-glucose as a glucose donor [5,6]. Once *S*. *cerevisiae* glycogenins initiate glycogen synthesis and form short-chain glucose oligomers on their Tyr^230^/Tyr^232^, glycogen synthases, along with the branching enzyme, produce a large, branched polysaccharide known as glycogen [1].

Glycogen is present in many organisms, from bacteria and archaea to eukaryotes, including humans [3]. Since glycogen is a primary intracellular reserve polymer, glycogen biosynthesis represents a major strategy to cope with subsequent starvation [6]. Prolonged starvation leads to the autophagic degradation of GGs inside lysosomes and a non-autophagic glycogen breakdown in the cytosol [6,7]. In mammals, the autophagic accumulation of GGs in lysosomes lacking the activity of acid α-glucosidase (GAA), the sole lysosomal glycogen hydrolase, contributes to the lysosomal storage disorder known as Pompe disease [8]. Recently, there has been some progress in understanding the autophagic trafficking of GGs to lysosomes: the delivery of GGs to lysosomes was suggested to be selective and called “glycophagy” to distinguish it from non-selective autophagy. However, glycophagy selectivity factors that package specifically GGs into autophagic vesicles (autophagosomes) remained unclear, except for the starch binding domain 1 (Stbd1) [9,10]. But even Stbd1 appeared to be liver-specific, questioning the selectivity of the process in the heart and skeletal muscles [11,12]. Nevertheless, what is clear is that autophagosomes play an important role in the transport of GGs from the cytosol to lysosomes [8].

As yeast in general (and *K*. *phaffii* in particular) is such a good autophagic model, we used *K*. *phaffii* cells to clarify the type of autophagy responsible for the degradation of GGs in eukaryotes. First, we developed *K*. *phaffii* as a model to study autophagic delivery and degradation of GGs under nitrogen starvation conditions. For this, we turned Glg1, the protein marker of GGs, into the Glg1-GFP autophagic reporter and measured the cytoplasm-to-vacuole delivery and degradation of GGs in autophagic, vacuolar, and glycogenin mutants. All our findings presented here unequivocally suggest that GGs are delivered to the vacuole for degradation by non-selective autophagy.

## 2. Materials and Methods

### 2.1. Strains and Plasmids

*K*. *phaffii* strains and plasmids used in this study are described in Table 1. All the plasmids, which were cloned using polymerase chain reaction (PCR) fragments, were verified by sequencing. Recipient strains were transformed with all the plasmids by electroporation [13]. All the plasmids were linearized with endonucleases of restriction (see below for details) before transformation for directed integration into the yeast genome.

The pRK22 plasmid with the Glg1-GFP expression cassette has a 500 bp promoter and an open reading frame (ORF) without the STOP codon of the *GLG1* gene. They were amplified by PCR using the genomic DNA of the wild-type (WT) strain and cloned as an XmaI-PstI fragment into the pRK1 vector [18]. The pNW11 plasmid is the site-directed mutagenesis product of pRK22 and encodes the Glg1-GFP (Y212F) variant of Glg1-GFP. The pNW10 plasmid was created using pRK22 and pRK35 plasmids. First, we built pRK35 containing the Pgk1-GFP expression cassette with a 500 bp promoter and ORF without the STOP codon of the *PGK1* gene. They were PCR amplified using the genomic DNA of the WT strain and cloned as a SpeI-PstI fragment into pRK1. Then, the promoter of *PGK1* on pRK35 was replaced with the *GLG1* promoter to make pNW10. For this, we substituted the pRK35 XmaI-PstI fragment carrying the entire *PGK1* locus with two fragments: (1) the XmaI-SpeI fragment with the *GLG1* promoter amplified by PCR from pRK22, and (2) the SpeI-PstI fragment with the *PGK1* ORF without STOP codon PCR amplified from pRK35.

All *HIS4* plasmids, including pRK22, pNW11, and pNW10, were linearized in the *HIS4* selection marker with EcoNI before transformation for their integration into *his4* locus of recipient strains. His^+^-transformants of strains with these plasmids were selected on SD+DOM-His plates (1.7 g/L yeast nitrogen base [YNB] without amino acids and ammonium sulfate, 5 g/L ammonium sulfate, 1.92 g/L drop-out mix synthetic minus histidine, 20 g/L dextrose, and 20 g/L agar) and screened for the expression of GFP fusion proteins by western blotting with mouse GFP antibodies (see Section 2.3 below).

The pNW9 plasmid with the *K. phaffii GLG1* (PAS_chr4_0847) deletion cassette was built by inserting the 1000 bp 3′-untranslated region (3′-UTR) of *GLG1* as a SpeI-NotI fragment into the Zeocin^R^ vector, pAP1, to create an intermediate plasmid, pNW8, and then by inserting the 1000 bp 5′-UTR of *GLG1* as a KpnI-SalI fragment into pNW8 to create pNW9. The *GLG1* deletion cassette containing the 5′-UTR of *GLG1*, the *Zeocin^R^* gene, and the 3′-UTR of *GLG1* was released from pNW9 by double digestion with KpnI and NotI before transformation. The *glg1* deletion mutant was selected as a Zeocin^R^-transformant of the WT strain on YPD+Zeocin plates (10 g/L yeast extract, 20 g/L peptone, 20 g/L dextrose, 20 g/L agar, and 100 mg/L Zeocin) and verified by PCR.

### 2.2. Iodine Staining for Glycogen

The glycogen content of yeast biomass was assessed by the iodine staining method [19], with modifications described in [20]. Briefly, strains were grown as patches on YPD plates (in duplicate) for 2 d at 30 °C. Then, plates were inverted over iodine crystals under the fume hood for 2 min. Patches synthesizing glycogen were stained brown.

### 2.3. Biochemical Analysis

For biochemical analysis, cells were grown in 1 mL of YPD medium (10 g/L yeast extract, 20 g/L peptone, and 20 g/L dextrose) for 1 d at 30 °C. Then, 1.5 ODs of cells were taken for analysis. Alternatively, 3 ODs of cells were washed twice with 1 mL of sterile 1× YNB solution (1.7 g/L YNB without amino acids and ammonium sulfate) and shifted to 3 mL of SD-N medium (1.7 g/L YNB without amino acids and ammonium sulfate, and 20 g/L dextrose) with the starting OD_600_ = 1. Then, 1 mL of cell culture was taken at 0 and 24 h time-points from SD-N medium. Protein lysates were prepared by trichloroacetic acid (TCA) precipitation [21] and assayed by western blotting. Samples were subjected to 10% SDS-PAGE after heating in a heat block for 10 min at 95 °C. Then, 15 µL of each sample was loaded into the gel and run at 115 V. Proteins were transferred from gels to nitrocellulose membranes for 1 h at 90 V. The membranes were stained with the Revert 700 total protein stain (926-11021, LI-COR Biosciences, Lincoln, NE, USA) for 5 min, de-stained for 1 min, and imaged in the Odyssey CLx imager (LI-COR Biosciences) in a 700 nm channel. Then, membranes were blocked in blocking solution (Tris-buffered saline with Tween-20 [TBS-T] containing 5% nonfat dry milk) for 1 h. After blocking, they were incubated with mouse GFP antibodies (11814460001, Roche Diagnostics, Mannheim, Germany; 1:2000 dilution in blocking solution) overnight at 4 °C. The next day, membranes were washed three times for 5 min each with 1× TBS-T. Then, they were incubated with IRDye 800CW goat anti-mouse antibodies (926-32210, LI-COR Biosciences; 1:15,000 dilution in TBS-T) for 1 h at room temperature and washed twice (5 min each) with 1× TBS-T and once (5 min) with 1× TBS. Finally, membranes were imaged in 700 and 800 nm channels, and images were quantified using the LI-COR Image Studio Lite v5.2 software. All experiments were performed at least twice in duplicate.

### 2.4. Fluorescence Microscopy

For fluorescence microscopy, cells were either grown only in YPD medium or grown in YPD medium, washed, and transferred to SD-N medium, as described in Section 2.3 (see above), with the following changes. After a fraction of cells was washed and transferred to SD-N medium (if necessary), the rest of the YPD cultures were stained for vacuolar lumen with a 10 mM stock solution of the CellTracker blue CMAC dye (C2110, Invitrogen, Eugene, OR, USA) in DMSO (the final concentration of the dye in the culture tube was 10 µM) for 30 min at 30 °C and imaged as a “0 min” or “0 h” time-point. The last 30 min of SD-N cultures was the incubation of cells with CMAC dye before imaging them as “30 min”, “9 h”, or “24 h” time-points. For imaging, cells at all time-points were immobilized in 1% low-melt agarose. For this, 2 µL of cell culture was placed on a slide and merged with 5 µL of 1% low-melt agarose at 37 °C placed on a coverslip. Cells in 5 non-overlapping fields of view were imaged on the Eclipse Ti2-E inverted microscope equipped with the CFI Plan Apochromat Lambda 100× oil objective and operated by the NIS Elements AR v5.20 software (Nikon Instruments Inc., Melville, NY, USA). All experiments were performed at least twice in duplicate.

### 2.5. Statistical Analysis

Statistical analysis was performed in the Microsoft Excel 2016 software on data obtained from at least three independent experiments in duplicate (N = 6). Statistical results are presented as the average ± standard deviation. The Student’s *t*-test (two-tailed distribution, two-sample unequal variance) was used to probe for statistical significance. Differences between two groups of samples were considered statistically significant if *p* < 0.05.

## 3. Results

### 3.1. Glg1-GFP Fusion Protein Is Functional in Glycogen Synthesis

**Figure 1:**
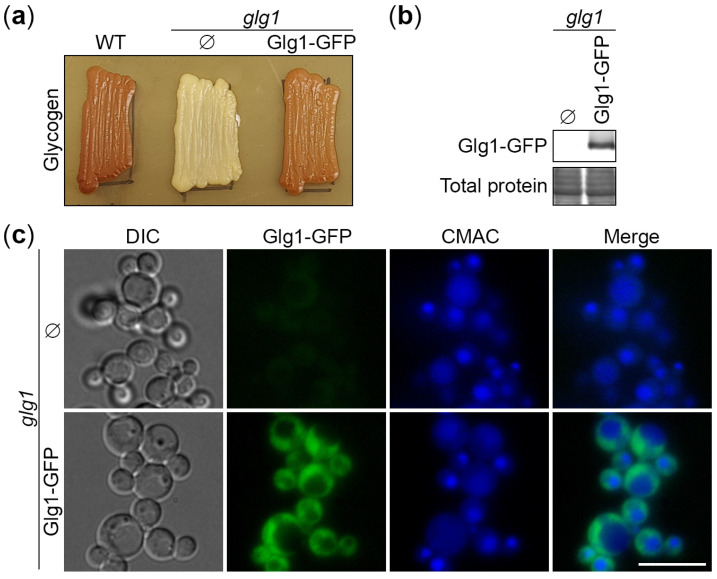
Glg1-GFP fusion protein is functional in glycogen synthesis. (**a**) Glycogen synthesis in *glg1* cells with the Glg1-GFP fusion protein. Patches of WT, empty *glg1*, and *glg1* rescued with the Glg1-GFP plasmid were grown for 2 d on the YPD plate and exposed to the vapor of iodine crystals for glycogen staining. Patches synthesizing glycogen were stained brown. (**b**) Expression of the Glg1-GFP fusion protein. The cells of the *glg1* mutant without and with the Glg1-GFP plasmid were grown for 1 d in YPD medium and studied for Glg1-GFP expression by immunoblotting with GFP antibodies. The total protein staining was used as a loading control. (**c**) Localization of the Glg1-GFP fusion protein. The same strains were grown for 1 d in YPD medium and studied for Glg1-GFP localization relative to the vacuole stained with CMAC dye. Scale bar, 10 µm.

To develop *K*. *phaffii* as a model for autophagic studies of GGs, we first tested if Glg1 is indeed a glycogenin responsible for the initiation of glycogen synthesis in this yeast. For this, we constructed the *glg1* deletion mutant by the gene replacement method (see Section 2.1 for details). Then, we studied glycogen synthesis in this strain on YPD plates using iodine staining for glycogen and found that *glg1* cells are indeed fully deficient in the production of glycogen relative to the WT strain (Figure 1a), as expected.

Next, we used Glg1, which is a *bona fide* marker of GGs because of its covalent linkage with glycogen, to create the Glg1-GFP fusion protein. For this, we generated the integrative plasmid, pRK22, with the P*_GLG1_*-*GLG1*-*GFP* expression cassette (see Section 2.1 for details). We transformed this plasmid into the *glg1* mutant and confirmed the expression of Glg1-GFP in YPD medium by western blotting using GFP antibodies (Figure 1b). Also, we studied the localization of Glg1-GFP relative to the vacuole stained with CMAC dye [22] by fluorescence microscopy (Figure 1c). This experiment in YPD medium showed a diffuse cytosolic localization of Glg1-GFP, as expected. Finally, we tested if Glg1-GFP could rescue glycogen synthesis in the *glg1* mutant and found that it was able to restore the production of glycogen in this glycogen-deficient strain (Figure 1a). This suggests that Glg1-GFP is functional (i.e., capable of self-glucosylation for the initiation of glycogen synthesis). As such, it can be used as a fluorescent marker of GGs.

### 3.2. Degradation of Glycogen Granules Depends on Autophagy and Vacuole

**Figure 2:**
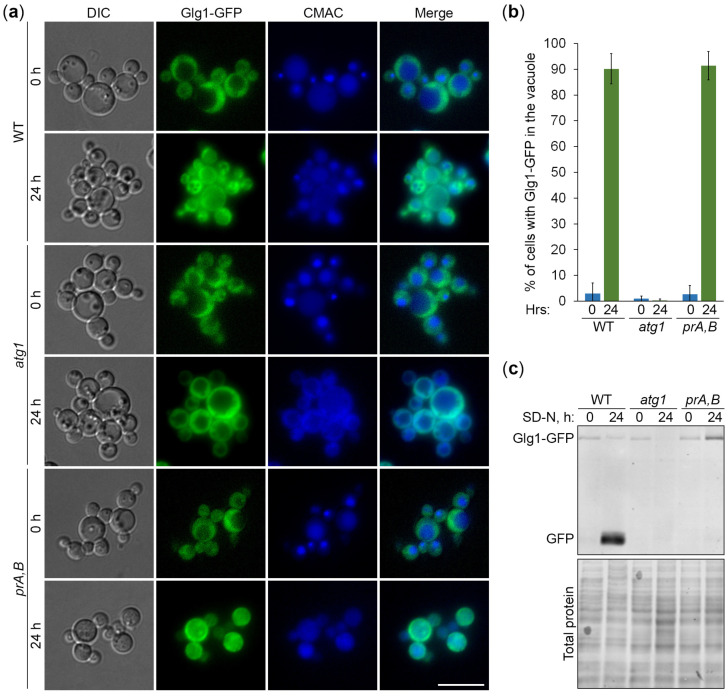
Degradation of glycogen granules depends on autophagy and vacuole. (**a**) Delivery of glycogen granules to the vacuole. The cells of WT, *atg1*, and *prA,B* (*pep4 prb1*) strains that express Glg1-GFP were grown for 1 d in YPD. First, a fraction of cells was transferred to SD-N at OD_600_ = 1 for 24 h. Then, the rest of the YPD cultures were stained for 30 min with CMAC and imaged as “0 h”. The last 30 min of SD-N cultures was incubation with CMAC before imaging them as “24 h”. Scale bar, 10 µm. (**b**) Quantification of images in (**a**). Displayed are averages and standard deviations. (**c**) Degradation of glycogen granules in the vacuole. The cells of WT, *atg1*, and *prA,B* strains that express Glg1-GFP were grown for 1 d in YPD. Then, a fraction of cells was transferred to SD-N at OD_600_ = 1. At 0 and 24 h, equal volumes of cultures (not equal biomass) were taken from SD-N for immunoblotting with GFP antibodies. The same membrane was also stained for total protein.

Nitrogen starvation in SD-N medium is a strong inducer of both selective and non-selective autophagic pathways in yeast. Previously, we developed *K*. *phaffii* as a model for nitrogen starvation-induced lipophagy, the selective autophagy of lipid droplets [23]. To study the mechanism of the GG degradation in the nitrogen-starved *K*. *phaffii*, we employed Glg1-GFP and mutants deficient in autophagy (*atg1* [15], for autophagy-related 1) and vacuolar proteolysis (*pep4 prb1* [17], also known as *prA,B* [23] because it is lacking proteinases A and B). In the first experiment, we monitored the delivery of Glg1-GFP-tagged GGs to the vacuole by fluorescence microscopy (Figure 2a). The cells of WT, *atg1*, and *prA,B* strains that express Glg1-GFP were grown for 1 d in YPD medium. First, a fraction of cells was transferred to SD-N medium at OD_600_ = 1 for 24 h. Then, the rest of the YPD cultures were stained for 30 min with CMAC and imaged as “0 h”. At this time-point, all the strains had Glg1-GFP localized in the cytosol, as expected (Figure 2a). The last 30 min of SD-N cultures was incubation with CMAC before imaging them as “24 h”. At that time-point, we observed a remarkable redistribution of Glg1-GFP from the cytosol to the vacuole in WT and *prA,B* strains, but not in the *atg1* mutant (Figure 2a). These results were confirmed by the quantification of the images (Figure 2b). The percentage of cells with Glg1-GFP inside the vacuole increased sharply after 24 h of nitrogen starvation for WT and *prA,B* strains, but not for *atg1*. Therefore, we concluded that *K*. *phaffii* GGs are delivered to the vacuole by autophagy. 

In the second experiment, we examined the degradation of Glg1-GFP-tagged GGs in the vacuole. The same strains were grown for 1 d in YPD. Then, a fraction of cells was transferred to SD-N at OD_600_ = 1. At 0 and 24 h, equal volumes of cultures were taken from SD-N for immunoblotting with GFP antibodies (Figure 2c). The immunoblotting showed that Glg1-GFP is efficiently processed to free GFP in nitrogen-starved WT, but not *atg1* and *prA,B* cells. The GFP processing assay is widely used in yeast autophagy studies because accumulation of proteolytically stable free GFP indicates delivery of the GFP fusion protein to the vacuole and its partial degradation therein. The lack of free GFP in *atg1* cells (Figure 2c) is consistent with the delivery block of GGs to the vacuole in *atg1* cells (Figure 2a), while the lack of free GFP in *prA,B* cells (Figure 2c) is consistent with the vacuolar degradation of Glg1 by proteinases A and B under nitrogen starvation conditions. Despite the highest total protein level (due to autophagy block), the *atg1* (24 h) lane had the lowest level of Glg1-GFP. The reason for much less Glg1-GFP in this lane might be increased solubility of glycogen in TCA and, consequently, much higher loss of Glg1-GFP during protein precipitation with TCA at this condition. Indeed, a part of the glycogen is acid-soluble [1], leading to the loss of a portion of Glg1-GFP covalently bound to such a glycogen during protein lysate preparation by TCA precipitation. Our microscopy showed that all GGs remained in the cytosol of the *atg1* strain at 24 h (Figure 2a). This might have made them more acid-soluble, preventing any TCA precipitation of the glycogen-bound Glg1-GFP. Collectively, in these experiments, we established that the degradation of GGs in nitrogen-starved *K*. *phaffii* cells depends on both autophagy and the vacuole.

### 3.3. Autophagy of Glycogen Granules Is Independent of Atg11

**Figure 3:**
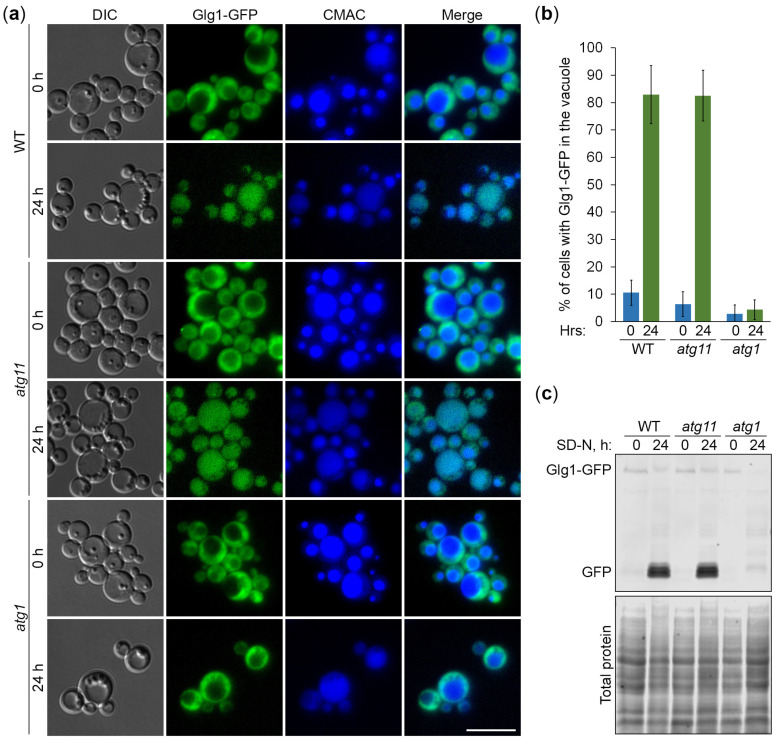
Autophagy of glycogen granules is independent of Atg11. (**a**) Delivery of glycogen granules to the vacuole. The cells of WT, *atg11*, and *atg1* strains that express Glg1-GFP were grown for 1 d in YPD. First, a fraction of cells was transferred to SD-N at OD_600_ = 1 for 24 h. Then, the rest of the YPD cultures were stained for 30 min with CMAC and imaged as “0 h”. The last 30 min of SD-N cultures was incubation with CMAC before imaging them as “24 h”. Scale bar, 10 µm. (**b**) Quantification of images in (**a**). Displayed are averages and standard deviations. (**c**) Degradation of glycogen granules in the vacuole. The cells of WT, *atg11*, and *atg1* strains that express Glg1-GFP were grown for 1 d in YPD. Then, a fraction of cells was transferred to SD-N at OD_600_ = 1. At 0 and 24 h, equal volumes of cultures (not equal biomass) were taken from SD-N for immunoblotting with GFP antibodies. The same membrane was also stained for total protein.

There are two major types of autophagy based on the specificity of cargo sequestration, selective and non-selective autophagy. Most selective autophagy pathways utilize the autophagic scaffold protein, Atg11, which is dispensable for non-selective autophagy [24]. To gain insight into the specificity of the GG autophagy in nitrogen-starved *K*. *phaffii*, we used the Glgl-GFP autophagic reporter and the *atg11* mutant. First, we monitored the delivery of GGs to the vacuole by microscopy (Figure 3a). The cells of WT, *atg11*, and *atg1* strains that express Glg1-GFP were grown in YPD, transferred to SD-N, and stained with CMAC, as above. At the 0 h time-point, Glg1-GFP was diffused in the cytosol in all the strains, as expected, but after 24 h in SD-N, Glg1-GFP moved from the cytosol to the vacuole in WT and *atg11* cells, but not in *atg1* cells (Figure 3a). The quantification of images confirmed these observations (Figure 3b). The percentage of cells with Glg1-GFP inside the vacuole increased dramatically after 24 h of nitrogen starvation for both WT and *atg11*, but not *atg1*. These results suggest that the autophagic delivery of GGs to the vacuole is an Atg11-independent process in *K*. *phaffii*. 

Next, we examined the degradation of GGs in the vacuole (Figure 3c). The same strains with Glg1-GFP were grown in YPD, transferred to SD-N, and sampled for immunoblotting, as above. The immunoblotting showed that Glg1-GFP is efficiently processed to free GFP in nitrogen-starved WT and *atg11* cells, but not *atg1* cells (Figure 3c). The presence of free GFP in *atg11* cells (Figure 3c) is consistent with the vacuolar delivery of GGs in *atg11* cells (Figure 3a) under nitrogen starvation conditions. Same as in Figure 2c, the total protein level is higher at 24 h than at 0 h in the *atg1* strain due to autophagy block (Figure 3c). Since GGs are restricted to the cytoplasm of the *atg1* strain at 24 h of nitrogen starvation (Figure 3a), they might have become more acid-soluble, preventing the appearance of Glg1-GFP, which is covalently bound to glycogen, in the TCA precipitate of proteins. Altogether, these results suggest that the autophagy of GGs in nitrogen-starved *K*. *phaffii* cells is independent of Atg11, raising the possibility that it might belong to a non-selective type of autophagy.

### 3.4. Autophagy of Glycogen Granules Is a Non-Selective Process

**Figure 4:**
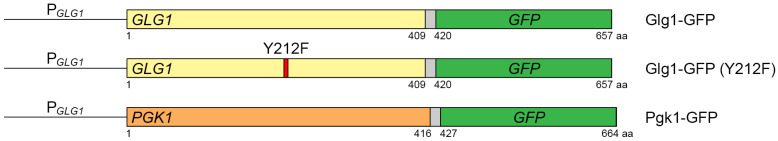
Cassettes for the expression of the glycogen granule-bound (Glg1-GFP) and cytosolic (Glg1-GFP [Y212F] and Pgk1-GFP) GFP fusion proteins under the same (*GLG1*) promoter.

**Figure 5:**
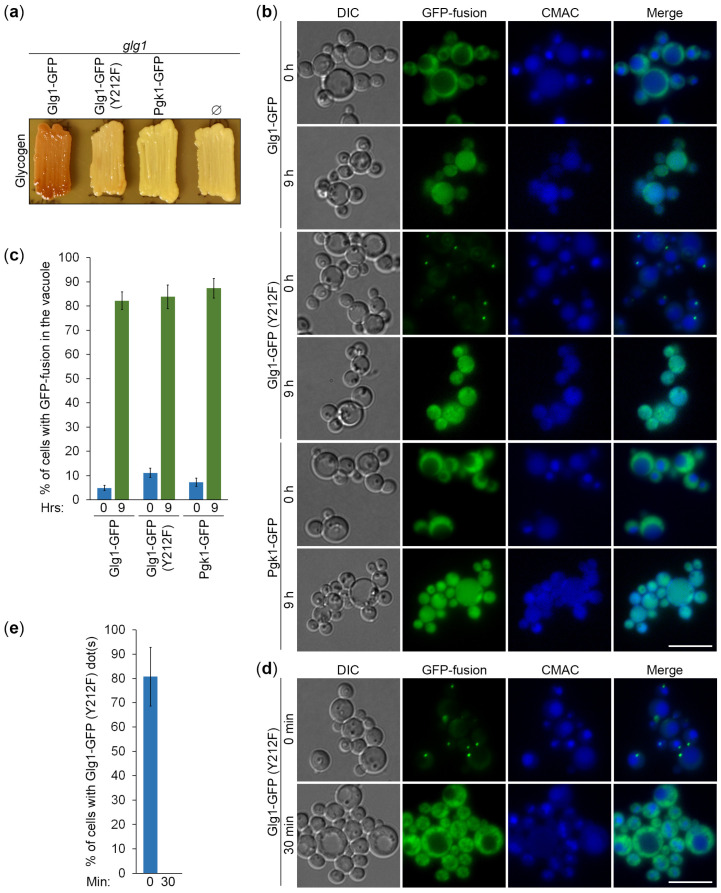
Glycogen granules are delivered to the vacuole by non-selective autophagy. (**a**) Glycogen synthesis in *glg1* cells with GFP fusion proteins. Patches of the indicated strains were grown for 2 d on the YPD plate and exposed to the vapor of iodine crystals for glycogen staining (stained brown). (**b**) Delivery of GFP fusion proteins to the vacuole. The *glg1* cells that express indicated GFP fusion proteins were grown for 1 d in YPD. First, a fraction of cells was transferred to SD-N at OD_600_ = 1 for 9 h. Then, the rest of the YPD cultures were stained for 30 min with CMAC and imaged as “0 h”. The last 30 min of SD-N cultures was incubation with CMAC before imaging them as “9 h”. Scale bar, 10 µm. (**c**) Quantification of images in (**b**). Displayed are averages and standard deviations. (**d**) Dissipation of Glg1-GFP (Y212F) dots before their delivery to the vacuole. The *glg1* cells that express Glg1-GFP (Y212F) were grown for 1 d in YPD. A fraction of cells was transferred to SD-N with CMAC for 30 min and imaged as “30 min” of nitrogen starvation. The rest of the YPD culture was stained for 30 min with CMAC and imaged as “0 min” of nitrogen starvation. Scale bar, 10 µm. (**e**) Quantification of images in (**d**). Displayed are averages and standard deviations.

To gain further insight into the specificity of Atg11-independent autophagy of GGs, we created two additional expression cassettes (Figure 4): (1) P*_GLG1_*-*GLG1^Y212F^*-*GFP* on the pNW11 plasmid, and (2) P*_GLG1_*-*PGK1*-*GFP* on the pNW10 plasmid (see Section 2.1 for details). The Pgk1-GFP fusion protein was expressed under the *GLG1* promoter. We did it to make Pgk1-GFP levels comparable with Glg1-GFP levels. The Tyr^212^ residue in *K. phaffii* Glg1 corresponds to the Tyr^230^/Tyr^232^ residue in *S. cerevisiae* Glg1/Glg2 (Figure S1). Therefore, the Y212F mutation in the Glg1-GFP fusion should create glycogenin that lacks a conserved tyrosine residue essential for self-glucosylation [5] and make it effectively a cytosolic (not GG-bound) protein, similar to the Pgk1-GFP fusion, which is a *bona fide* cytosolic marker and an established reporter for non-selective autophagy [25]. We transformed pNW11 and pNW10 plasmids into *glg1* cells and confirmed that the mutated Glg1-GFP (Y212F) variant is indeed unable to rescue glycogen synthesis in these glycogen-deficient cells, like Pgk1-GFP, but in contrast to the non-mutated Glg1-GFP variant (Figure 5a). As such, these new cytosolic GFP fusions can help us to clarify the type of autophagy of GGs.

To compare the delivery of GGs and cytosol to the vacuole, *glg1* strains that express GG-bound (Glg1-GFP) and cytosolic (Glg1-GFP [Y212F] and Pgk1-GFP) GFP fusions were grown in YPD, transferred to SD-N, and stained with CMAC. At 0 h of nitrogen starvation, Glg1-GFP and Pgk1-GFP were diffused in the cytosol, as expected, but Glg1-GFP (Y212F) accumulated on the dot-like structures (Figure 5b). These Glg1-GFP (Y212F) dots were present in 81% of cells at 0 min of nitrogen starvation (Figure 5d,e). However, they quickly dissolved, making the cytosolic localization of this GFP fusion evident just 30 min into nitrogen starvation before the delivery of the protein to the vacuole. As such, we were not interested in their nature because the Glg1-GFP (Y212F) fusion behaved essentially as a cytosolic protein after 30 min of nitrogen starvation. After 9 h in SD-N, all three GFP fusions moved from the cytosol to the vacuole (Figure 5b). Quantification of images showed that vacuolar deliveries of GG-bound Glg1-GFP and cytosolic Glg1-GFP (Y212F) were comparable and not more efficient than a non-selective vacuolar delivery of Pgk1-GFP (Figure 5c). Therefore, the *K*. *phaffii* GGs and cytosolic Glg1-GFP (Y212F) are delivered to the vacuole by non-selective autophagy.

**Figure 6:**
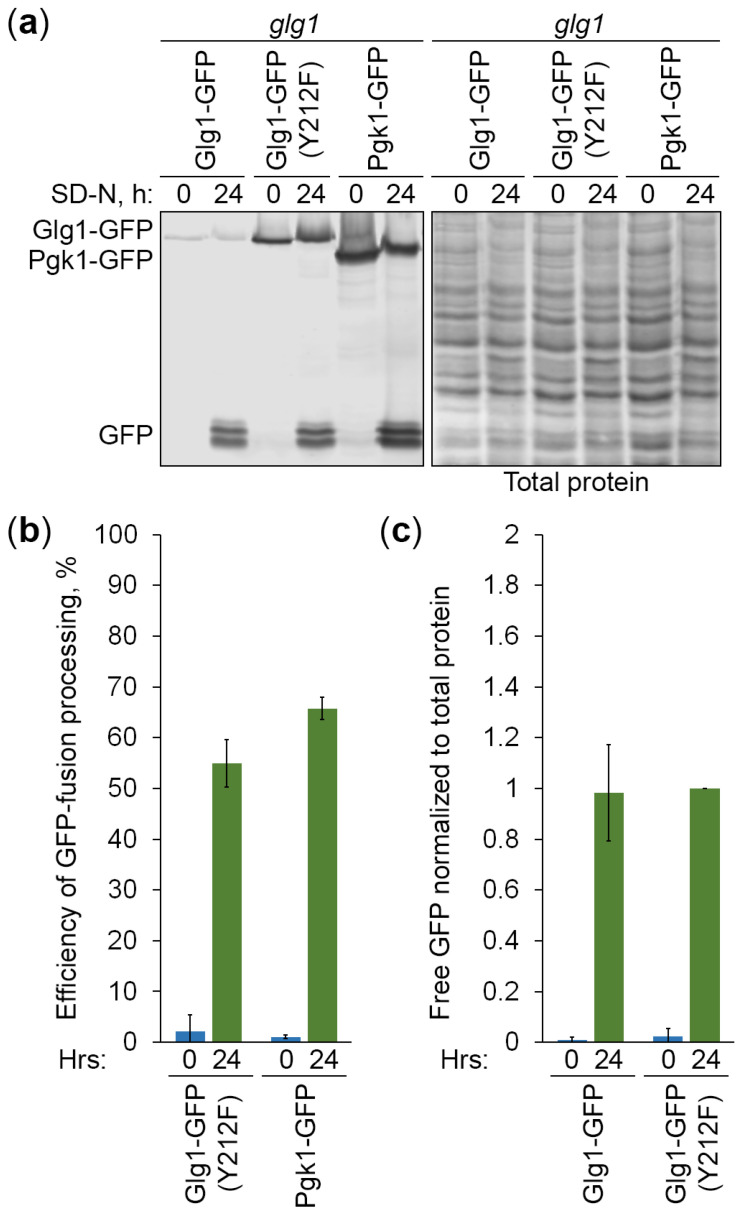
Glycogen granules are degraded in the vacuole by non-selective autophagy. (**a**) Processing of GFP fusion proteins in the vacuole. The *glg1* cells that express indicated GFP fusion proteins were grown for 1 d in YPD. Then, a fraction of cells was transferred to SD-N at OD_600_ = 1. At 0 and 24 h, equal volumes of cultures (not equal biomass) were taken from SD-N for immunoblotting with GFP antibodies. The same membrane was also stained for total protein, which was used in the quantification below. (**b**,**c**) Quantifications of immunoblotting in (**a**). Displayed are averages and standard deviations.

To compare the vacuolar degradation of GGs and cytosol, the above strains that express GG-bound and cytosolic GFP fusions were grown in YPD, transferred to SD-N, and sampled for immunoblotting. Despite the same promoter being used to express GFP fusions, immunoblotting with GFP antibodies showed that protein levels were different (Figure 6a). They increased in order from Glg1-GFP to Glg1-GFP (Y212F) to Pgk1-GFP. Higher levels of Glg1-GFP (Y212F) than Glg1-GFP are most probably due to a better TCA precipitation of the non-glucosylated (mutated) variant of the protein than the variant of the protein that is covalently bound to GGs. Indeed, a sizable portion of GGs is acid-soluble [1], leading to the loss of GG-bound Glg1-GFP during protein lysate preparation by TCA precipitation. As such, the efficiency of GFP fusion processing (GFP to [GFP + GFP fusion] ratio) cannot be accurately assessed for the Glg1-GFP fusion because the level of Glg1-GFP is underestimated. At the same time, the efficiencies of GFP fusion processing were comparable for two other GFP fusion proteins (Figure 6b). This suggests that cytosolic Glg1-GFP (Y212F) is degraded by non-selective autophagy, like Pgk1-GFP, in agreement with the comparable delivery of two proteins to the vacuole (Figure 5c).

Even though we could precipitate only minute amounts of GG-bound Glg1-GFP, its delivery to the vacuole and vacuolar processing released free GFP that was precipitated by TCA, the same as GFP released from other GFP fusions. Therefore, free GFP levels liberated from different GFP fusions could be compared after their normalization to total protein levels. We excluded the free GFP from Pgk1-GFP fusion from this analysis, because Pgk1-GFP had higher expression levels, which caused higher free GFP levels. The free GFP levels from the other two GFP fusions normalized to corresponding total protein levels were nearly identical (Figure 6c). Therefore, we concluded that the GG-bound Glg1-GFP is degraded by non-selective autophagy, like cytosolic Glg1-GFP (Y212F), in agreement with comparable efficiencies of their delivery to the vacuole (Figure 5c).

## 4. Discussion

In this study, we developed *K. phaffii* yeast as a first, simple model to study the autophagy of GGs and addressed the type of autophagy responsible for their elimination under nitrogen starvation conditions. To monitor the delivery of GGs to the vacuole and their degradation therein, we took advantage of the covalent linkage between GG and glycogenin (Glg1) protein and created the Glg1-GFP fusion protein. First, we proved that Glg1-GFP fusion is functional (i.e., able to synthesize glycogen in the *glg1* mutant, which is fully devoid of this storage polysaccharide) (Figure 1). Then, we showed that the degradation of GGs in the nitrogen-starved *K. phaffii* cells depends on autophagy and the vacuole (Figure 2). However, it does not depend on the most common autophagic selectivity factor, Atg11 (Figure 3), questioning the selectivity of the GG autophagy. To clarify the type of GG autophagy, we compared the efficiencies of vacuolar delivery and degradation for GGs and cytosol, which is known to be degraded by non-selective autophagy. For this, we used the established autophagic reporter for cytosol, Pgk1-GFP. Also, we disrupted the ability of Glg1-GFP to generate GGs by introducing the Y212F mutation, making this GFP fusion a cytosolic protein (Figure 4). The experiments with these additional autophagic reporters and a non-mutated Glg1-GFP showed that GGs, similar to the cytosol, are delivered to the vacuole (Figure 5) and degraded there (Figure 6) by non-selective autophagy. Therefore, the autophagy of GGs is a non-selective process in the nitrogen-starved *K. phaffii* cells.

It will be interesting to see if the same is true in *K. phaffii* cells under other experimental conditions, e.g., during gradual carbon starvation in the stationary phase of growth. Previously, we showed that nitrogen starvation lipophagy and stationary phase lipophagy have distinct molecular mechanisms [23]. Therefore, we cannot exclude the possibility that the autophagy of GGs is a selective process in *K. phaffii* cells under other circumstances, like carbon starvation. Moreover, studies in nitrogen-starved cells of other yeast species, e.g., *S. cerevisiae*, must be conducted to clarify if non-selective autophagy of GGs is a generic response of yeasts to nitrogen starvation. 

It would be even more important to re-evaluate the autophagy of GGs in mammalian cells because it contributes to Pompe disease, which is a lysosomal storage disorder with overaccumulation of GGs inside lysosomes [8]. The accumulation of glycogen in the lysosomes of nerve and muscle cells causes cardiac, musculoskeletal, and respiratory complications and ultimately results in premature death [26,27]. Although the hallmark pathology of Pompe disease is lysosomal glycogen accumulation, the precise mechanism of autophagic delivery of GGs to lysosomes remains unknown, especially in cardiac and skeletal muscles. Understanding this mechanism is important for discovering novel therapeutic targets for Pompe disease. Our study clarified the type of autophagy responsible for the degradation of GGs in the *K. phaffii* yeast. This is important because even though there is evidence of selectivity for the delivery of GGs to lysosomes in mouse liver, where it is mediated by Stbd1 protein, Stbd1 does not contribute to this process in cardiac and skeletal muscles [11,12]. Hence, it is possible that in non-hepatic mammalian tissues, the autophagy of GGs is a non-selective process, like in *K. phaffii* cells.

## 5. Conclusions

In conclusion, we established the *K. phaffii* yeast as a simple model for the autophagy of GGs and elucidated that it is a non-selective process under nitrogen starvation conditions. Our study raises the possibility that in non-hepatic mammalian tissues, GGs might also be degraded by non-selective autophagy and not by glycophagy.

## Figures and Tables

**Table 1:** *K. phaffii* strains and plasmids used in this study.

Mutant	Strain	Background	Genotype and Plasmid	Source
WT	PPY12h	PPY12h	*arg4 his4*	[14]
WT	SRK147	PPY12h	*his4*::pRK22 *(*P*_GLG1_-GLG1-GFP, HIS4)*	This study
*atg1*	R12	GS115	*atg1-1*::*Zeocin^R^ his4*	[15]
*atg1*	SRK149	R12	*his4*::pRK22 *(*P*_GLG1_-GLG1-GFP, HIS4)*	This study
*atg11*	R8	GS115	*atg11-2*::*Zeocin^R^ his4*	[16]
*atg11*	SNW7	R8	*his4*::pRK22 *(*P*_GLG1_-GLG1-GFP, HIS4)*	This study
*glg1*	SNW49	PPY12h	∆*glg1*::*Zeocin^R^* (pNW9)	This study
*glg1*	SNW65	SNW49	*his4*::pRK22 *(*P*_GLG1_-GLG1-GFP, HIS4)*	This study
*glg1*	SNW80	SNW49	*his4*::pNW11 *(*P*_GLG1_-GLG1^Y212F^-GFP, HIS4)*	This study
*glg1*	SNW82	SNW49	*his4*::pNW10 *(*P*_GLG1_-PGK1-GFP, HIS4)*	This study
*pep4 prb1*	SMD1163	GS115	*pep4 prb1 his4*	[17]
*pep4 prb1*	SRK151	SMD1163	*his4*::pRK22 *(*P*_GLG1_-GLG1-GFP, HIS4)*	This study

## Data Availability

The original contributions presented in the study are included in the article, further inquiries can be directed to the corresponding author.

## Supplementary Materials

The following supporting information can be downloaded at https://www.mdpi.com/article/10.3390/cells13060467/s1. Figure S1: Multiple sequence alignment of *K. phaffii* Glg1 and its orthologues in *Homo sapiens* and *S. cerevisiae*. Reference [28] is cited in Figure S1 legend.
