# Supplementary material for: Glycogen Granules Are Degraded by Non-Selective Autophagy in Nitrogen-Starved Komagataella phaffii
Source: Cells. 2024 Mar 7;13(6):467. doi: 10.3390/cells13060467 (PMC10969688; doi:10.3390/cells13060467)
Supplement: Supplementary file 1 [file cells-13-00467-s001.zip › cells-2848652-supplementary.pdf]

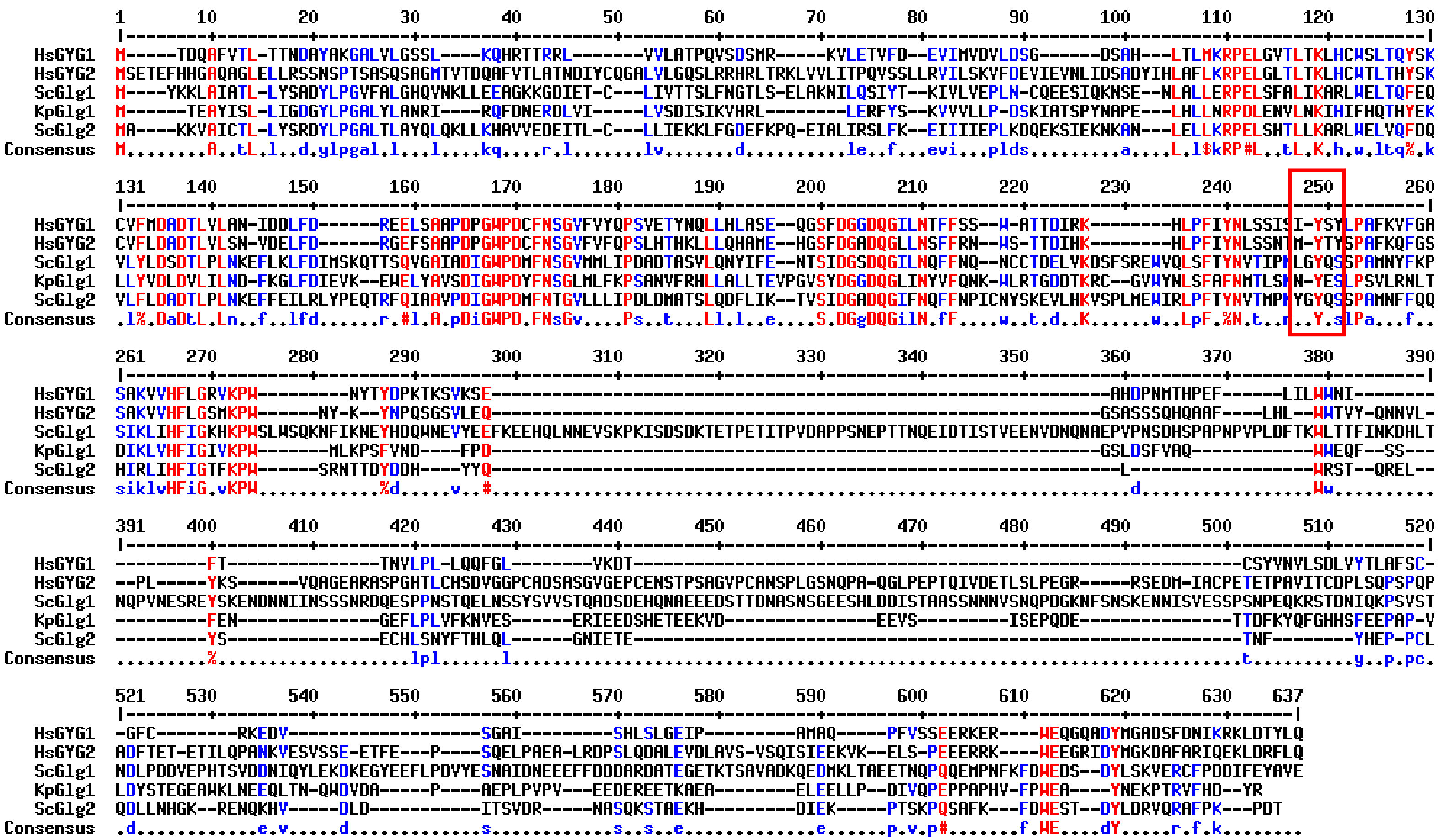

Figure S1. Multiple sequence alignment of *K. phaffii* Glg1 and its orthologues in *Homo sapiens* and *S. cerevisiae*. HsGYG1, *H. sapiens* glycogenin 1 (P46976); HsGYG2, *H. sapiens* glycogenin 2 (O15488); ScGlg1, *S. cerevisiae* glycogenin-like gene 1 (YKR058W); KpGlg1, *K. phaffii* glycogenin-like gene 1 (XP_002494295.1); ScGlg2, *S. cerevisiae* glycogenin-like gene 2 (YJL137C). Color code of amino acid residues: red – high consensus, blue – low consensus, black – neutral. The conserved tyrosine (Y) residue, which forms the glucose-1-*O*-tyrosyl linkage, is framed in the red rectangle. The alignment was generated using MultAlin software [28]. Alignment parameters: Dayhoff – 8 – 0 (symbol comparison table – gap penalty at opening – gap penalty at extension).
